# Relationships Between Health-Related Quality of Life and Speech Perception in Bimodal and Bilateral Cochlear Implant Users

**DOI:** 10.3389/fpsyg.2022.859722

**Published:** 2022-04-28

**Authors:** Nadav Brumer, Elizabeth Elkins, Jake Hillyer, Chantel Hazlewood, Alexandra Parbery-Clark

**Affiliations:** ^1^Auditory Research Laboratory, Swedish Neuroscience Institute, Seattle, WA, United States; ^2^College of Medicine, University of Arizona, Phoenix, AZ, United States

**Keywords:** cochlear implant, health related quality of life, speech perception, hearing loss, bimodal, bilateral, Glasgow Benefit Inventory, Nijmegen Cochlear Implant Questionnaire

## Abstract

**Purpose:**

Previous studies examining the relationship between health-related quality of life (HRQoL) and speech perception ability in cochlear implant (CI) users have yielded variable results, due to a range of factors, such as a variety of different HRQoL questionnaires and CI speech testing materials in addition to CI configuration. In order to decrease inherent variability and better understand the relationship between these measures in CI users, we administered a commonly used clinical CI speech testing battery as well as two popular HRQoL questionnaires in bimodal and bilateral CI users.

**Methods:**

The Glasgow Benefit Inventory (GBI), a modified five-factor version of the GBI (GBI-5F), and the Nijmegen Cochlear Implant Questionnaire (NCIQ) were administered to 25 CI users (17 bimodal and 8 bilateral). Speech perception abilities were measured with the AzBio sentence test in several conditions (e.g., quiet and noise, binaural, and first-ear CI only).

**Results:**

Higher performance scores on the GBI general subscore were related to greater binaural speech perception ability in noise. There were no other relationships between the GBI or NCIQ and speech perception ability under any condition. Scores on many of the GBI-5F factors were substantially skewed and asymmetrical; therefore, correlational analyses could not be applied. Across all participants, binaural speech perception scores were greater than first-ear CI only scores.

**Conclusion:**

The GBI general subscore was related to binaural speech perception, which is considered the everyday listening condition of bimodal and bilateral CI users, in noise; while the more CI-specific NCIQ did not relate to speech perception ability in any listening condition. Future research exploring the relationships between the GBI, GBI-5F, and NCIQ considering bimodal and bilateral CI configurations separately is warranted.

## Introduction

Cochlear implants (CIs) significantly improve quality of life (QoL) and speech perception abilities for individuals with severe to profound hearing loss (Gaylor et al., 2013; Mosnier et al., 2015). Currently, speech perception scores are the primary outcome measures utilized for quantifying CI benefit in adult users; however, there is a growing movement to further quantify CI benefit with QoL measures. This is because while objective measures of CI benefit, such as speech perception tests, are important for evaluating CI performance, CIs also influence other aspects of a patient’s life, such as self-esteem and socializing, that are not always captured by traditional objective measures. In general, QoL measures capture important information regarding the subjective wellbeing of a patient at a given point in time. On the other hand, health-related quality of life (HRQoL) measures are specific to certain aspects of QoL affected by health conditions, such as hearing loss, or medical procedures, such as cochlear implantation. As such, HRQoL measures may be more sensitive to differences in CI benefit compared to general QoL measures (Krabbe et al., 2000; Hirschfelder et al., 2008; Sladen et al., 2017). As HRQoL measures are increasingly used with CI recipients in clinical settings, it is important to understand any relationships between these HRQoL metrics and the traditional metric of CI-aided speech perception scores.

The use of HRQoL measures in addition to speech perception scores to monitor CI benefit is growing in popularity (Services, U.D.O.H.A.H., 2011). HRQoL questionnaires that are commonly used with CI patients are the Glasgow Benefit Inventory (GBI; Robinson et al., 1996) adapted for CI users (Ho et al., 2009) and the Nijmegen Cochlear Implant Questionnaire (NCIQ; Hinderink et al., 2000). The GBI was initially developed to be a post-intervention outcome measure for medical treatments including surgical procedures, and is scored on a scale from −100 to +100, meaning that clinicians can identify if there has been an overall improvement (closer to +100) or worsening (closer to −100) of QoL post-intervention. The GBI consists of 18 questions, and scoring includes a total score as well as three subscores: general, social support, and physical health. One strength of the GBI is that it addresses the direct success of CI implantation with questions regarding whether or not an individual would undergo the procedure again or recommend it to others, providing additional information for capturing subjective benefit of a CI. However, recent work highlights the need to explore the construct validity of the GBI subscores; specifically, which questions are designated to each subscore (Browning et al., 2021). A confirmatory factor analysis performed by Browning et al. (2021) found that the original three subscore model of the GBI was a poor fit for data from 4,799 otolaryngologic patient responses and that the total score and general subscore contained a large number of heterogeneous questions that do not converge on any one construct. Browning et al. (2021) further identified three questions that were either redundant or not pertinent to otolaryngologic intervention in this group (e.g., question nine centered around job opportunities and was the most frequently unanswered of the 18 total questions in the GBI in this population). As such, Browning recommended that, for an otolaryngologic patient population, the general subscore of the original GBI be split into three additional subscores (QoL, self-confidence, and social involvement) and that three less relevant questions be removed from the original 18 questions. This modified GBI questionnaire was renamed the five-factor Glasgow Benefit Inventory (GBI-5F), which has 15 rather than 18 questions and contains a total of five subscores.

A second popular clinical questionnaire is the NCIQ (Hinderink et al., 2000). Unlike the GBI and GBI-5F, the NCIQ was specifically created for CI users and is scored on a Likert scale from 1–5 with transformed scores ranging from 0 (very poor) to 100 (optimal). In addition to the 5-point Likert system, the NCIQ includes a sixth “not applicable” option to all questions, which may be helpful for distinguishing between what domains are not affected by cochlear implantation and which are simply less relevant to a certain population of CI users. Unlike the brief 18 question GBI, the NCIQ consists of 60 questions centered around several physical, psychological, and social domains related to CI use. While the NCIQ is considerably longer than the GBI, this allows for a theoretically more comprehensive assessment of CI outcomes which may increase sensitivity to various clinical changes (Hinderink et al., 2000). Indeed, the NCIQ has been shown to be sensitive to pre- to post-implantation performance change which distinguishes itself from retrospective HRQoL questionnaires, such as the GBI (Straatman et al., 2014; McRackan et al., 2018).

Relating subjective HRQoL questionnaire responses to clinical objective speech perception ability for CI recipients has yielded mixed results. The GBI total and general subscores have been shown to relate to CI-aided speech perception ability in specific instances, such as listening for sentences in quiet (Palmer et al., 1999; Hillyer et al., 2019); however, similar relationships are not apparent when different CI age groups or speech perception materials are included (i.e., sentences versus words). For example, correlations between GBI scores and speech perception of monosyllabic words in quiet existed for younger (<55 years) but not older (≥55 years) CI users (Vermeire et al., 2005). Conversely, Sorrentino et al. (2020) found the opposite effect, with relationships observed between GBI scores and speech perception ability in quiet for three different test stimuli (i.e., disyllabic words, sentences, and question comprehension) in an older (≥65 years) but not in a younger (≤50 years) group of CI users. Meanwhile, Forli et al. (2019) observed no correlations between GBI score and speech perception ability of Italian disyllabic words in quiet or noise, regardless of age group (42–80 years).

Inconsistent relationships between speech perception ability and NCIQ scores have also been observed. CI benefit measured with pre-operative NCIQ scores and 12  months post-operative NCIQ scores related to gains in speech perception of disyllabic words (Mosnier et al., 2015) and monosyllabic words (Sladen et al., 2017). This finding has been further supported by associations between NCIQ subdomains and speech perception abilities of words in quiet (Capretta and Moberly, 2016) and sentences in noise (Olze et al., 2012; Capretta and Moberly, 2016). Conversely, Hirschfelder et al. (2008) found no relationship between CI-aided speech perception ability of monosyllabic words in noise and NCIQ scores. Similarly, our previous research found no association between NCIQ scores and speech perception ability of sentences in quiet (Hillyer et al., 2019), possibly because participants were considered high-performing CI users, thus limiting variability in CI-aided speech perception performance. Consistent with these variable results, a meta-analysis of 13 studies examining HRQoL questionnaires, including the NCIQ, found negligible to weak but significant correlations between HRQoL measures and speech perception ability of sentences in quiet and noise (McRackan et al., 2018).

In addition to the large variation of participant’s age groups and speech stimuli used, another potential explanation for mixed relationships observed between HRQoL and speech perception ability is that previous studies have included participants using a variety of CI configurations. Previous research has often included a variety of combinations of CI users, including unilateral [i.e., one CI with no contralateral amplification], bimodal [i.e., one CI (electric signal) with a contralateral hearing aid (acoustic signal) and/or bilateral [i.e., two CIs (two electric signals)] users, with users experiencing conditions in both quiet and noise. Indeed, Olze et al. (2012) evaluated unilateral CI users only, while other works evaluated primarily unilateral CI and bimodal users (Mosnier et al., 2015; Forli et al., 2019) or a combination of unilateral CI, bimodal and bilateral users (Sanchez-Cuadrado et al., 2015; Capretta and Moberly, 2016; McRackan et al., 2018; Hillyer et al., 2019; Sorrentino et al., 2020). Additionally, some studies do not delineate between bimodal or bilateral CI users (Vermeire et al., 2005; Hirschfelder et al., 2008; Sladen et al., 2017). Therefore, it is challenging to generalize how HRQoL measures may relate to speech perception abilities in CI users when CI configurations included in studies are variable.

The first goal of the present study was to examine how HRQoL score relates to speech perception ability in bimodal and bilateral CI users. By focusing on bimodal and bilateral CI users, we decrease some of the inherent variability in group performance that is observed when unilateral CI users are included. Our second goal was to examine speech perception ability with a first-ear CI only and binaural configuration across all participants. We administered the GBI, the GBI-5F, and the NCIQ, and had participants complete CI-aided speech perception tasks in four conditions: (1) first-ear CI only configuration in quiet, (2) first-ear CI only configuration in noise, (3) binaural (i.e., two bilateral CI’s or HA and CI) configuration in quiet, and (4) binaural configuration in noise. Clinical speech scores were used in this study as the overarching goal of this research was to improve understanding of the relationship between clinical CI speech understanding and HRQoL measures. Given that similar data is collected by audiologists across the United States as standard of care, this work has the potential for meaningful clinical translation and interpretation by audiologists providing care to this patient population. We predicted that higher speech perception scores would relate to higher HRQoL scores for the GBI, GBI-5F, and NCIQ. However, we also predicted that HRQoL domains less impacted by speech perception abilities (e.g., physical health) would be less related to speech performance. We also predicted that participants would have higher speech perception scores in the binaural condition relative to the first-ear CI only condition.

## Materials and Methods

### Participants

Twenty-five (14 females, 11 males) experienced CI users (>6 months CI listening experience, *M* = 63.4 months, SD = 31.86 months, range of 14–145 months) with bimodal (*n* = 17) and bilateral (*n* = 8) configurations, between the ages of 52 and 82 years (*M* = 67.28, SD = 10.09) were recruited from the patient pool at the Center for Hearing and Skull Base Surgery at The Swedish Neuroscience Institute in Seattle, Washington. Experienced CI users were recruited because maximum comfortable levels and threshold levels are optimally achieved after 6 months of use and programming (Gajadeera et al., 2017). Inclusion criteria required participants to have no recorded symptoms or diagnosis of dementia, no report of cognitive decline, and no history of congenital or pre-lingual hearing loss. All participants were native speakers of English, had at least a high school education, and demonstrated normal IQ scores (*M* = 107.24.11, SD = 7.85), as measured by the Test of Non-verbal Intelligence—4th Edition (TONI-4; Brown et al., 2010). All testing procedures were approved by the Swedish Medical Center Institutional Review Board (#SWD56152-14) and participants provided informed written consent. All speech testing was conducted in a booth, and all questionnaires were completed in a clinic room at the Swedish Neuroscience Institute in Seattle, WA, United States. All subjects completed all measures except for five who did not complete the NCIQ (*n* = 20; 15 bimodal, 5 bilateral for this measure).

### CI-Aided Speech Perception Testing (AzBio)

All participants completed speech perception testing in both quiet and noise conditions, with both first-ear CI only (i.e., no HA or second CI) and binaural configurations (i.e., either CI + HA or CI + CI) in a randomized manner. The speech perception test material chosen was the AzBio Sentence Test (Spahr et al., 2012), comprised of recordings of 20 sentences spoken by two male and two female talkers. Sentences range from 4 to 10 words, spoken by one talker at a time in a conversational style with minimal contextual cues (e.g., “She missed a week of work and nobody noticed”). All words presented are keywords for scoring purposes. Speech testing was administered in a sound-proof booth, with internal dimensions of 2.74 m x 2.82 m. Speech stimuli were presented at 60 dB SPL from a loudspeaker (GN Otometrics Astera Sound Field Speakers) at 0 degrees azimuth, 2 m from the participant, who was instructed to repeat back what they heard. The noise condition, presented at a signal-to-noise ratio (SNR) of +8 dB, included additional 10-talker babble from the same loudspeaker. All speech testing and scoring was performed by a CI audiologist as part of each participant’s routine audiologic care and represents the data that was used for clinical decision making for programming and treatment. AzBio speech scores were reported as a percentage (%) of total words correctly repeated, with higher scores indicating better performance.

### Health-Related Quality of Life Measures

**The Glasgow Benefit Inventory (GBI)** is based on a five-point Likert scale that ranges from one, signifying a large change for the worse, to five, signifying a large change for the better, with a score of three signifying no change. Total scores (i.e., the sum responses to 18 questions which is then scaled and averaged) range from −100 (i.e., maximum worsening of overall health status post-intervention) to +100 (maximum improvement of overall health status post-intervention). The total composite score and general subscore were also calculated with question 9 excluded given the evidence of question 9 (i.e., “job opportunities”) being potentially less relevant to an otolaryngological population (Browning et al., 2021) or older population as in our study.

**The GBI-5F** (Browning et al., 2021) is a revised version of the original GBI, with five subscores or factors instead of three. These five subscores are: QoL, self-confidence, support, social involvement, and general health, as well as a sixth total score. The general health and support subscores are identical to the original GBI physical and social support subscores, respectively. The GBI-5F removed questions 9, 10, and 14 from the original GBI based on relative importance to otolaryngologic intervention, redundancy, and the fact that questions 10 and 14 did not fit into any of the new constructs or factors created in the new GBI-5F.

**The Nijmegen Cochlear Implant Questionnaire (NCIQ)** is a HRQoL questionnaire specific to CI users (Hinderink et al., 2000). The 60-item scale consists of three domains: physical, psychological, and social. Within each domain are various subdomains consisting of 10 questions each. The physical domain consists of three subdomains: *basic sound perception*, *advanced sound perception,* and *speech production*; the psychological domain has one subdomain: *self-esteem*, and the social domain consists of two subdomains: *activity limitations* and *social interactions*. The NCIQ is scored on a Likert scale from 1 to 5 and transformed so that 1 = 0, 2 = 25, 3 = 50, 4 = 75, and 5 = 100 These scores were then summed together and then divided by the number of completed questions, with scores ranging from 0 (poor) to 100 (optimal). Higher scores indicate better overall health-related quality of life.

### Statistical Analyses

Statistical analyses were completed using SPSS Version 28 (IBM Corp, 2021). Prior to analysis, normality of data was evaluated using Shapiro–Wilk tests. HRQoL and speech perception scores were analyzed using Pearson correlations, as well as paired sample t-test comparisons to assess the effects of CI configuration (i.e., first-ear CI only vs. binaural). For Pearson correlations between HRQoL scores and speech perception performance, a power analysis conducted utilizing G ^*^ Power indicated that there was an 80% chance of detecting a medium to large effect for a sample size that ranged between 9 and 29 (*α* = 0.05). For paired sample *t*-tests, G ^*^ Power indicated an 80% chance of detecting a medium to large effect with a sample size that ranged from 15 to 34 (*α* = 0.05). Spearman Rho correlations were used when needed for measures that were not normally distributed. Semi-partial correlations were employed to examine the relationship between speech perception ability and HRQoL measures given that age was related to speech perception ability in all conditions, except for first-ear CI only in quiet, but not any HRQoL scores. Semi-partial correlations are similar to partial correlations but are used to examine the relationship between two variables while taking into account a covariate that is related to only one of these variables, such as age. Several GBI-5F scores were significantly asymmetrical and skewed (i.e., the distribution of the data was skewed toward the maximum possible score). Skewness was defined as any point beyond the established Fisher’s skewedness coefficient (*SK_F_*) range of −1.96 and + 1.96 (Pett, 2015), meaning the GBI-5F could not be subjected to correlational analyses and thus only descriptive statistics are reported. Ceiling and floor effects for the GBI-5F were defined as significant if ≥15% of participants scored the highest or lowest possible score for a given subscore (Gulledge et al., 2019). Bonferroni corrections were applied where appropriate. All reported statistics reflect two-tailed significance values.

## Results

### HRQoL Descriptive Statistics

**Figure 1:**
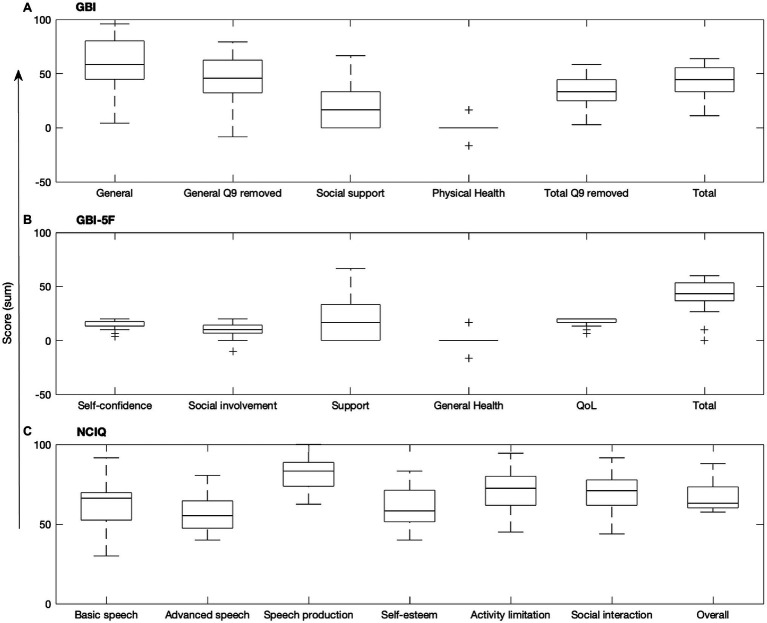
Boxplots of the **(A)** GBI, **(B)** GBI-5F, and **(C)** NCIQ. Central mark indicates median, bottom and top edges of box indicate 25th and 75th percentiles, whiskers extend to minimum and maximum points, and plus signs indicate outliers. *Q9, question 9*.

The GBI total score and general subscore (calculated with and without question 9) were interrelated (all *r* ≤ 0.991, *p* ≤ 0.001), but the GBI social support and physical health subscores were not related to the GBI total score or other subscores (all *ρ* ≤ 0.211, *r* ≤ 0.965; Bonferroni adjusted *α* = 0.008; see Table 1). For the GBI-5F, three out of five of the subscores were substantially asymmetrical, with QoL (*kurtosis* = 2.90, *skewness* = −1.69, *SK_F_* = −3.64), social involvement (*kurtosis* = 1.87, *skewness* = −1.25, *SK_F_* = −2.70), and the total score (*kurtosis* = 2.29, *skewness* = −1.358, *SK_F_* = −2.93) being significantly skewed. Additionally, 56% and 28% of scores on the QoL and self-confidence subscores, respectively, were significantly at ceiling. In comparison, 4% of support scores, 0% of general health scores, 8% of social involvement scores, and 0% of total scores were at ceiling. No floor effects were observed for any subscores. On the general health subscore, 80% of participants indicated no change or had a “0” score since the CI surgery (see Figure 1). NCIQ total score was related to all NCIQ subscores (advanced sound perception, speech production, self-esteem, activity limitation, and social interaction; all *ρ* ≤ 0.757, *p* ≤ 0.002) except for basic sound perception (*ρ* = 0.560, *p* = 0.010; Bonferroni adjusted *α* = 0.007). Only NCIQ activity limitation and social interaction subscores of the NCIQ were interrelated (*r* = 0.737, *p* < 0.001); no other relationships between NCIQ subdomains were observed (all *r* ≤ 0.493, *p* ≤ 0.951; see Table 2).

**Table 1:** Intercorrelations between the GBI.

	Total	Total Q9 removed	General subscore	General subscore Q9 removed	Social support	Physical health
Total	–	*r* **= 0.970,***p* **< 0.001**	*r* **= 0.969,***p* **< 0.001**	*r* **= 0.962,***p* **< 0.001**	*ρ* = −0.145, *p* = 0.490	*ρ* = 0.145, *p* = 0.489
Total Q9 removed	–	–	*r* **= 0.936,***p* **< 0.001**	*r* **= 0.940,***p* **< 0.001**	*ρ* = −0.104, *p* = 0.621	*ρ* = 0.211, *p* = 0.311
General subscore	–	–	**–**	*r* **= 0.991,***p* **< 0.001**	*ρ* = −0.326, *p* = 0.112	*ρ* = 0.033, *p* = 0.875
General subscore Q9 removed	–	–	–	–	*ρ* = −0.308, *p* = 0.134	*ρ* = 0.014, *p* = 0.948
Social support	–	–	–	–	–	*ρ* = −0.009, *p* = 0.965
Physical health	–	–	–	–	–	–

Q9, question 9; Bold = significant with Bonferroni correction.

**Table 2:** Intercorrelations between the NCIQ.

	Basic sound perception	Advanced sound perception	Speech production	Self-esteem	Activity limitation	Social interaction	Overall
Basic sound perception	–	*r* = 0.387, *p* = 0.083	*r* = 0.208, *p* = 0.379	*r* = 0.447, *p* = 0.048	*r* = 0.195, *p* = 0.410	*r* = 0.348, *p* = 0.133	*ρ* = 0.560, *p* = 0.010
Advanced sound perception	–	–	*r* = 0.099, *p* = 0.679	*r* = 0.335, *p* = 0.149	*r* = 0.493, *p* = 0.027	*r* = 258, *p* = 0.271	***ρ* = 0.700,***p* **< 0.001**
Speech production	–	–	–	*r* = 0.147, *p* = 0.536	*r* = 0.048, *p* = 0.840	*r* = −0.015, *p* = 0.951	***ρ* = 0.309,***p* **= 0.185**
Self-esteem	–	–	–	–	*r* = 0.557, *p* = 0.011	*r* = 0.391, *p* = 0.088	***ρ* = 0.722,***p* **< 0.001**
Activity limitation	–	–	–	–	–	*r* **= 0.737,***p* **< 0.001**	***ρ* = 0.757,***p* **= 0.001**
Social interaction	–	–	–	–	–	–	***ρ* = 0.659,***p* **= 0.002**
Overall	–	–	–	–	–	–	–

Bold = significant with Bonferroni correction.

### Speech Perception and HRQoL Questionnaires

No relationships between speech perception ability with a first-ear CI only configuration in quiet and any GBI scores were observed (all *ρ* ≤ 0.482, *p* ≤ 0.846). There were significant semi-partial correlations between binaural speech perception performance in noise and the GBI general subscore with question 9 included (*r* = 0.463, *p* = 0.016) and question 9 excluded (*r* = 0.442, *p* = 0.024; see Table 3). No semi-partial relationships between speech perception scores and any other GBI scores were observed (all *r* ≤ 0.428, *p* ≤ 0.591; see Table 3). GBI-5F scores were not subjected to correlational analyses due to asymmetry and skewedness. There were no relationships between speech perception performance scores and any NCIQ scores (all *r* ≤ 0.190, *p* ≤ 0.972; see Table 4).

**Table 3:** Relationships between AzBio and GBI scores.

GBI measures	AzBio first-ear CI only in quiet	AzBio first-ear CI only in noise	AzBio binaural quiet	AzBio binaural noise
Total	*ρ* = 0.407, *p* = 0.044	*r* = 0.380, *p* = 0.055	*r* = 0.203, *p* = 0.321	*r* = 0.371, *p* = 0.062
Total Q9 removed	*ρ* = 0.385, *p* = 0.057	*r* = 0.370, *p* = 0.063	*r* = 0.132, *p* = 0.522	*r* = 0.293, *p* = 0.148
General	*ρ* = 0.337, *p* = 0.099	*r* = 0.406, *p* = 0.037	*r* = 0.268, *p* = 0.181	*r* **= 0.463,***p* **= 0.016**
General Q9 removed	*ρ* = 0.354, *p* = 0.082	*r* = 0.428, *p* = 0.029	*r* = 0.249, *p* = 0.221	*r* **= 0.442,***p* **= 0.024**
Social support	*ρ* = 0.041, *p* = 0.846	*r* = −0.110, *p* = 0.601	*r* = −0.291, *p* = 0.146	*r* = −0.405, *p* = 0.038
Physical Health	*ρ* = 0.132, *p* = 0.530	*r* = −0.201, *p* = 0.336	*r* = −0.085, *p* = 0.671	*r* = −0.166, *p* = 0.405

Q9, question 9; Bold = significant with Bonferroni correction.

**Table 4:** Relationships between AzBio and NCIQ scores.

NCIQ measures	AzBio first-ear CI only in quiet	AzBio first-ear CI only in noise	AzBio binaural in quiet	AzBio binaural in noise
Basic sound perception	*ρ* = 0.380, *p* = 0.187	*r* = −0.038, *p* = 0.758	*r* = −0.156 *p* = 0.530	*r* = −0.087, *p* = 0.768
Advanced speech perception	*ρ* = 0.130, *p* = 0.585	*r* = 0.201, *p* = 0.482	*r* = 0.011, *p* = 0.940	*r* = 0.176, *p* = 0.411
Speech production	*ρ* = −0.070, p = 0.768	*r* = 0.035 *p* = 0.822	*r* = −0.068, *p* = 0.769	*r* = −0.1488, *p* = 0.515
Self-esteem	*ρ* = −0.356, *p* = 0.123	*r* = −0.464, *p* = 0.056	*r* = −0.237, *p* = 0.277	*r* = −0.257, *p* = 0.213
Activity limitation	*ρ* = 0.037, *p* = 0.878	*r* = −0.030, *p* = 0.877	*r* = −0.033, *p* = 0.897	*r* = −0.113, *p* = 0.656
Social interaction	*ρ* = 0.180, *p* = −0.045	*r* = −0.082, *p* = 0.703	*r* = 0.108, *p* = 0.653	*r* = −0.063, *p* = 0.812
Overall	*ρ* = −0.045, *p* = 0.850	*r* = −0.096, *p* = 0.677	*r* = −0.104, *p* = 0.674	*r* = −0.116, *p* = 0.642

### First-Ear CI Only Versus Binaural Speech Perception Analyses

**Figure 2:**
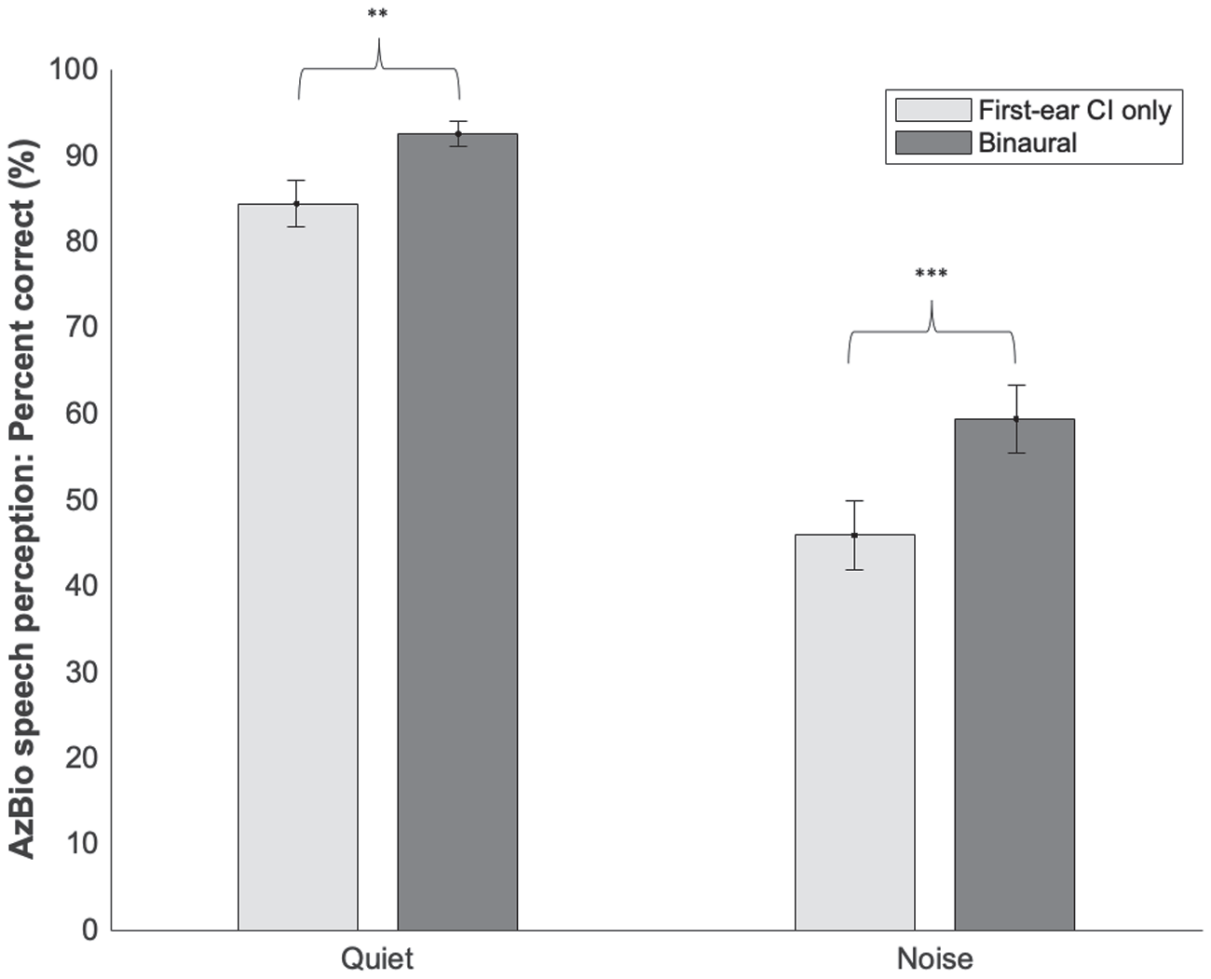
Means and SD’s of first-ear CI only and binaural speech perception scores in quiet and noise. Significant differences were noted between a first-ear CI only and binaural configuration in both quiet and noise. ^**^≤0.01, ^***^≤0.001.

Across all participants, a paired sample *t*-test comparing speech perception ability in quiet with the first-ear CI only (*M* = 84.36, SD = 13.59) versus the binaural configuration in quiet (*M* = 92.44, SD = 7.43) demonstrated better performance scores with a binaural configuration [*t*(24) = −3.58, *p* = 0.002; see Figure 2]. Speech perception ability in noise with a binaural configuration (*M* = 59.36, SD = 19.84) was better than with a first-ear CI only configuration [*M* = 45.84, SD = 20.19; *t*(24) = −4.28, *p* < 0.001]. The average increase in speech perception score going from a first-ear CI only to a binaural configuration was 8.1% points in quiet (SD = 11.28) and 13.52% points in noise (SD = 15.81).

## Discussion

The primary goal of this study was to explore how subjective ratings of CI benefit measured with the GBI, GBI-5F, and NCIQ related with objective CI outcomes measures of speech perception ability in bimodal and bilateral CI users. With respect to our first goal, the general subscore of the GBI related with speech perception ability in noise with CI users in a binaural configuration. However, no other relationships with the GBI, GBI-5F, or NCIQ were observed. Regarding our second goal, across all participants (bimodal and bilateral combined) higher speech perception ability was observed with the respective binaural configuration over a first-ear CI only configuration.

In the current study, we found that the GBI general subscore related with CI-aided speech perception ability in noise, only when a binaural configuration was used. Indeed, speech perception in noise is generally considered more reflective of everyday living conditions given that there is a certain level of noise present in our daily listening environment (Schafer et al., 2007; Wu et al., 2018). While we saw a positive correlation between CI speech in noise perception and the GBI general subscore, the GBI also contains a variety of questions aimed at various domains of health, such as physical health. In line with our second prediction regarding domains less relevant to speech perception ability, we found that GBI subscores calculated from questions not related to speech perception ability demonstrated no relationship with CI-aided speech perception performance. For example, the physical health subscore, which contains questions centered around changes in medications or frequency of illnesses that have occurred since cochlear implantation, was not correlated with any speech measures, indicating physical health may not be a strong indicator of CI benefit in this population. Indeed, in our study, 93% of participants reported no change on the three questions contributing to the physical health subscore, with 100% of participants reporting no change for question 12: *Since you had cochlear implant surgery, do you catch colds or infection more or less often?* Similarly, on average, 68% of participants reported no change on the three questions in the social support subscore, with 96% of participants reporting no change for question 11: *Since your cochlear implant surgery are there more or fewer people who really care about you?* The physical health subscore, followed by the social support subscore, were the subscores closest to an overall average of zero, further indicating that participants experienced the least amount of change in these domains after receiving a CI. These results suggest that these subscores and the questions included in them were less relevant to our group of CI users in terms of overall benefit in HRQoL from their CI. These results are consistent with previous studies where the least amount of benefit post-implantation was measured *via* the physical health subscore followed by the social support subscore (Lassaletta et al., 2006; Straatman et al., 2014; Sanchez-Cuadrado et al., 2015; Amin et al., 2021).

While the GBI general subscore did relate to speech perception abilities in this study, the total composite score did not, perhaps due to questions included that may have been less relevant to the patient population in this study. The GBI general subscore does not include questions from either the physical or social subscore, whereas the total composite score includes questions from both. In our study, it could be argued that speech perception ability may not have related to the total subscore for precisely this reason, in that it contained a larger number of questions less pertinent to CI outcomes. Similar to Browning et al. (2021), four subjects in our study indicated that question 9 was not relevant to them and therefore chose “no change” but would have preferred a “not applicable” option. This is understandable due to the nature of question 9 which discusses employment opportunities which may be less of a consideration for older individuals who were retired, did not work or participants who had not experienced recent job transitions. To assess the impact of this question we created an alternate score for any subscore that included this question (i.e., the total and general subscore). As expected, when removing this question from the total and general subscore, we did find significant differences between the average scores with and without this question removed. However, removal of question 9 did not alter the correlational relationships with CI speech perception, indicating that this question alone did not have a significant impact on the GBI’s general subscores sensitivity to CI speech ability. Browning et al. (2021) found that removal of question 9 from 3,436 participants that had completed the question made no material difference in terms of the average total score, although the N of the study was much greater and the differences between the general subscore with and without question 9 removed were not reported.

Another method to reorganize the GBI into potentially more meaningful constructs by grouping more homogenous questions and removing those less pertinent was developed by Browning et al. (2021). By employing this scoring method named the GBI-5F, we were able to explore whether these new constructs which are embedded within the original GBI may be more reflective of HRQoL for CI users. However, our results demonstrated that in this CI population, GBI-5F subscores were substantially skewed compared to the original GBI (see Figure 1). For example, 78% of participants reported a 5 (i.e., much better) in response to questions regarding change in QoL, suggesting that QoL greatly increased following cochlear implantation, but also that this subset of questions may not be specific enough for differentiating between degrees of benefit for this patient population if a majority of participants chose the maximum value. Given that the original GBI and GBI-5F general health and support subscores are identical with both scoring methods, no change to the restricted range was noted in the GBI-5F. The ceiling effects and restricted range observed within the new GBI-5F domains may be because the GBI-5F removed several questions from the original GBI (9, 10, and 14), thus reducing its sensitivity in this population. However, it may also be because the GBI-5F was developed for a broader range of otolaryngologic patients rather than a specific subset of that population, (i.e., CI users). Given the unique nature of CI users within the sphere of otolaryngologic intervention, additional work is needed before any clinical recommendations for GBI-5F use in this population can be determined.

While we saw a positive correlation between the GBI general subscores and CI speech measures in noise, this was not apparent for CI speech measures and the NCIQ. These results, however, are consistent with previous studies (Capretta and Moberly, 2016; Hillyer et al., 2019) and a meta-analysis which indicated low to negligible correlations between the NCIQ and speech perception abilities in both quiet and noise (McRackan et al., 2018). This may be because the NCIQ was developed to identify CI benefit by comparing pre-operative and post-operative scores (Hinderink et al., 2000), whereas our study examined post-implantation scores only. Indeed, studies that have demonstrated a relationship with NCIQ speech domains (i.e., basic sound perception, advanced sound perception, and speech production) and speech perception, have analyzed the change in speech perception in fixed pre- and post-implantation time ranges (Hirschfelder et al., 2008; Olze et al., 2012; Häußler et al., 2019). As such, the NCIQ appears to be more clinically applicable to CI-aided speech perception abilities when evaluating benefit through pre-post implantation scores rather than relating them to post-implantation scores alone.

The second goal of our study explored the differences within bimodal and bilateral CI user groups in terms of CI-aided speech perception ability. As expected in our third prediction, we found that across all participants (bimodal and bilateral combined) CI-aided speech perception scores in quiet and noise were higher with a binaural configuration versus a first-ear CI only configuration. This was evidenced by an 8.1% point increase on average in quiet and a 13.5% point increase in noise after adding a second CI for bilateral users or a HA for bimodal users, suggesting that binaural amplification was beneficial for speech perception performance. These results align with previous research demonstrating a speech perception benefit when moving from unilateral CI to a bimodal (Ching et al., 2004, 2008; Iwaka et al., 2004; Morera et al., 2005; Schafer et al., 2007; Illg et al., 2014; Farinetti et al., 2015; Hua et al., 2017) or bilateral CI (Gantz et al., 2002; Ramsden et al., 2005; Litovsky et al., 2006; Schafer et al., 2007; Buss et al., 2008).

One limitation of our study was a small sample size, specifically with regards to bilateral CI users (8 and 5 for the GBI and NCIQ respectively). While our sample size is not atypical of research surrounding bilateral users (Potts and Litovsky, 2014; Gifford et al., 2015; Moberly et al., 2018) comparing speech and HRQoL measures between bimodal and bilateral CI users can only be considered preliminary. In this study, these preliminary results indicate that bimodal and bilateral CI demonstrated essentially equivalent performance on CI-aided speech perception ability with a first-ear CI only configuration. However, bilateral CI users had an average binaural speech perception score of 97.13% in quiet and 69.88% in noise, while bimodal CI users had an average binaural speech perception score of 90.24% in quiet and 54.41% in noise. These results would appear in line with the meta-analysis of bimodal and bilateral CI users by Schafer et al. (2011), in which bilateral CI users had a slight but significant advantage in binaural performance over bimodal users in noise. It may be that more difficult speech perception tests, such as speech in background noise, are more likely to reveal a binaural benefit in bilateral CI users as evidenced by Wackym et al. (2007) in which the greatest and most consistent binaural benefit was observed with sentences presented in noise, followed by words presented in quiet. These results are also consistent with previous research that has seen more binaural benefit for bilateral CI users relative to a unilateral configuration, in noise than in quiet, for both word and sentence-level materials (Ramsden et al., 2005; Schafer et al., 2007). Future work with a larger sample size is needed to address the potential differences in binaural benefit between bimodal and bilateral CI.

## Conclusion

Our work demonstrates that the GBI general subscore related to speech perception ability in bimodal and bilateral everyday listening conditions unlike the total, physical or social support subscores, while the CI-specific NCIQ did not relate to speech perception ability in any domains. The GBI-5F had significant limitations when applied to this patient population due to skewedness, and therefore, recommendations for clinical applicability in CI users would be premature. Given the variability in the current literature due to the wide variety of speech testing materials and HRQoL questionnaires used, future research should aim to explore the relationships between clinical measures and these HRQoL questionnaires in each of the CI configurations separately.

## Data Availability Statement

The datasets presented in this article are not readily available because the Auditory Research Laboratory is part of a hospital system that does not allow for data sharing due to patient privacy requirements. Requests to access the datasets should be directed to the corresponding author.

## Ethics Statement

The studies involving human participants were reviewed and approved by Swedish Medical Center Institutional Review Board (#SWD56152-14). The patients/participants provided their written informed consent to participate in this study.

## Author Contributions

NB, AP-C, and JH participated in data collection. NB and AP-C created figures and tables and completed statistical analysis of data. NB, EE, and AP-C wrote manuscript. CH and JH contributed to editing the manuscript. All authors contributed to the article and approved the submitted version.

## Conflict of Interest

The authors declare that the research was conducted in the absence of any commercial or financial relationships that could be construed as a potential conflict of interest.

## Publisher’s Note

All claims expressed in this article are solely those of the authors and do not necessarily represent those of their affiliated organizations, or those of the publisher, the editors and the reviewers. Any product that may be evaluated in this article, or claim that may be made by its manufacturer, is not guaranteed or endorsed by the publisher.

## References

[ref1] Amin N. Wong G. Nunn T. Jiang D. Pai I. (2021). The outcomes of Cochlear implantation in elderly patients: A single United Kingdom center experience. Ear Nose Throat J. 100(Suppl. 5), 842S–847S. doi: 10.1177/0145561320910662, PMID: 32204619

[ref2] Brown L. Sherbenou R. J. Johnsen S. K. (2010). Test of Nonverbal Intelligence: TONI-4. TX: Pro-Ed, Inc.

[ref3] Browning G. G. Kubba H. Whitmer W. M. (2021). Revised 15-item Glasgow benefit inventory with five factors based on analysis of a large population study of medical and surgical otorhinolaryngological interventions. Clin. Otolaryngol. 46, 213–221. doi: 10.1111/coa.13649, PMID: 32949108

[ref4] Buss E. Pillsbury H. C. Buchman C. A. Pillsbury C. H. Clark M. S. Haynes D. S.. (2008). Multicenter US bilateral MED-EL Cochlear implantation study: speech perception over the first year of use. Ear Hear. 29, 20–32. doi: 10.1097/AUD.0b013e31815d7467, PMID: 18091099

[ref5] Capretta N. R. Moberly A. C. (2016). Does quality of life depend on speech recognition performance for adult cochlear implant users? Laryngoscope 126, 699–706. doi: 10.1002/lary.25525, PMID: 26256441

[ref6] Ching T. Y. Incerti P. Hill M. (2004). Binaural benefits for adults who use hearing aids and cochlear implants in opposite ears. Ear Hear. 25, 9–21. doi: 10.1097/01.AUD.0000111261.84611.C8, PMID: 14770014

[ref7] Ching T. Y. C. Massie R. Van Wanrooy E. Rushbrooke E. Psarros C. (2008). Bimodal fitting or bilateral implantation? Cochlear Implants Int. 10, 23–27. doi: 10.1002/cii.381 19067435

[ref9] Farinetti A. Roman S. Mancini J. Baumstarck-Barrau K. Meller R. Lavieille J. P.. (2015). Quality of life in bimodal hearing users (unilateral cochlear implants and contralateral hearing aids). Eur. Arch. Otorhinolaryngol. 272, 3209–3215. doi: 10.1007/s00405-014-3377-8, PMID: 25373837

[ref10] Forli F. Lazzerini F. Fortunato S. Bruschini L. Berrettini S. (2019). Cochlear implant in the elderly: results in terms of speech perception and quality of life. Audiol. Neurootol. 24, 77–83. doi: 10.1159/000499176, PMID: 31117068

[ref11] Gajadeera E. A. Galvin K. L. Dowell R. C. Busby P. A. (2017). The change in electrical stimulation levels During 24 months Postimplantation for a large cohort of adults using the nucleus^®^ Cochlear implant. Ear Hear. 38, 357–367. doi: 10.1097/aud.0000000000000405, PMID: 28166089

[ref12] Gantz B. J. Tyler R. S. Rubinstein J. T. Wolaver A. Lowder M. Abbas P.. (2002). Binaural Cochlear implants placed during the same operation. Otol. Neurotol. 23, 169–180. doi: 10.1097/00129492-200203000-00012, PMID: 11875346

[ref14] Gaylor J. M. Raman G. Chung M. Lee J. Rao M. Lau J.. (2013). Cochlear implantation in adults: a systematic review and meta-analysis. JAMA Otolaryngol. Head Neck Surg. 139, 265–272. doi: 10.1001/jamaoto.2013.1744 23429927

[ref15] Gifford R. H. Driscoll C. L. Davis T. J. Fiebig P. Micco A. Dorman M. F. (2015). A within-subjects comparison of bimodal hearing, bilateral cochlear implantation, and bilateral cochlear implantation with bilateral hearing preservation: high-performing patients. Otology and neurotology: official publication of the American Otological Society, American Neurotology Society [and] European academy of. Otol. Neurotol. 36, 1331–1337. doi: 10.1097/MAO.0000000000000804, PMID: 26164443 PMC4746722

[ref16] Gulledge C. M. Smith D. G. Ziedas A. Muh S. J. Moutzouros V. Makhni E. C. (2019). Floor and ceiling effects, time to completion, and question burden of PROMIS CAT domains Among shoulder and knee patients undergoing nonoperative and operative treatment. JBJS Open Access 4:15. doi: 10.2106/JBJS.OA.19.00015, PMID: 32043052 PMC6959920

[ref17] Häußler S. M. Knopke S. Wiltner P. Ketterer M. Gräbel S. Olze H. (2019). Long-term benefit of unilateral Cochlear implantation on quality of life and speech perception in bilaterally deafened patients. Otol. Neurotol. 40, e430–e440. doi: 10.1097/mao.0000000000002008, PMID: 30870378

[ref18] Hillyer J. Elkins E. Hazlewood C. Watson S. D. Arenberg J. G. Parbery-Clark A. (2019). Assessing cognitive abilities in high-performing Cochlear implant users. Front. Neurosci. 12:1056. doi: 10.3389/fnins.2018.01056, PMID: 30713488 PMC6346679

[ref19] Hinderink J. B. Krabbe P. F. Van Den Broek P. (2000). Development and application of a health-related quality-of-life instrument for adults with cochlear implants: the Nijmegen cochlear implant questionnaire. Otolaryngol. Head Neck Surg. 123, 756–765. doi: 10.1067/mhn.2000.108203, PMID: 11112975

[ref20] Hirschfelder A. Grabel S. Olze H. (2008). The impact of cochlear implantation on quality of life: the role of audiologic performance and variables. Otolaryngol. Head Neck Surg. 138, 357–362. doi: 10.1016/j.otohns.2007.10.019, PMID: 18312885

[ref21] Ho E. C. Monksfield P. Egan E. Reid A. Proops D. (2009). Bilateral bone-anchored hearing aid: impact on quality of life measured with the Glasgow benefit inventory. Otol. Neurotol. 30, 891–896. doi: 10.1097/MAO.0b013e3181b4ec6f 19692937

[ref22] Hua H. Johansson B. Magnusson L. Lyxell B. Ellis R. J. (2017). Speech recognition and cognitive skills in bimodal Cochlear implant users. J. Speech Lang. Hear. Res. 60, 2752–2763. doi: 10.1044/2017_JSLHR-H-16-0276, PMID: 28885638

[ref8] IBM Corp (2021). IBM SPSS Statistics for Macintosh, Version 28.0. Armonk, NY: IBM Corp.

[ref23] Illg A. Bojanowicz M. Lesinski-Schiedat A. Lenarz T. Büchner A. (2014). Evaluation of the bimodal benefit in a large cohort of Cochlear implant subjects using a contralateral hearing aid. Otol. Neurotol. 35, e240–e244. doi: 10.1097/mao.0000000000000529, PMID: 25058838

[ref24] Iwaka T. Matsushiro N. Mah S.-R. Sato T. Yasuoka E. Yamamoto K.-I.. (2004). Comparison of speech perception between monaural and binaural hearing in cochlear implant patients. Acta Otolaryngol. 124, 358–362. doi: 10.1080/00016480310000548a, PMID: 15224853

[ref25] Krabbe P. F. Hinderink J. B. van den Broek P. (2000). The effect of cochlear implant use in postlingually deaf adults. Int. J. Technol. Assess. Health Care 16, 864–873. doi: 10.1017/s0266462300102132 11028141

[ref26] Lassaletta L. Castro A. Bastarrica M. de Sarria M. J. Gavilan J. (2006). Quality of life in postlingually deaf patients following cochlear implantation. Eur. Arch. Otorhinolaryngol. 263, 267–270. doi: 10.1007/s00405-005-0987-1, PMID: 16025257

[ref27] Litovsky R. Parkinson A. Arcaroli J. Sammeth C. (2006). Simultaneous bilateral Cochlear implantation in adults: A multicenter clinical study. Ear Hear. 27, 714–731. doi: 10.1097/01.aud.0000246816.50820.42, PMID: 17086081 PMC2651401

[ref28] McRackan T. R. Bauschard M. Hatch J. L. Franko-Tobin E. Droghini H. R. Nguyen S. A.. (2018). Meta-analysis of quality-of-life improvement after cochlear implantation and associations with speech recognition abilities. Laryngoscope 128, 982–990. doi: 10.1002/lary.26738, PMID: 28731538 PMC5776066

[ref29] Moberly A. C. Harris M. S. Boyce L. Vasil K. Wucinich T. Pisoni D. B.. (2018). Relating quality of life to outcomes and predictors in adult cochlear implant users: are we measuring the right things? Laryngoscope 128, 959–966. doi: 10.1002/lary.26791, PMID: 28776711 PMC6192249

[ref30] Morera C. Manrique M. Ramos A. Garcia-Ibanez L. Cavalle L. Huarte A.. (2005). Advantages of binaural hearing provided through bimodal stimulation via a cochlear implant and a conventional hearing aid: A 6-month comparative study. Acta Otolaryngol. 125, 596–606. doi: 10.1080/00016480510027493, PMID: 16076708

[ref31] Mosnier I. Bebear J. P. Marx M. Fraysse B. Truy E. Lina-Granade G.. (2015). Improvement of cognitive function after cochlear implantation in elderly patients. JAMA Otolaryngol. Head Neck Surg. 141, 442–450. doi: 10.1001/jamaoto.2015.129, PMID: 25763680

[ref32] Olze H. Grabel S. Forster U. Zirke N. Huhnd L. E. Haupt H.. (2012). Elderly patients benefit from cochlear implantation regarding auditory rehabilitation, quality of life, tinnitus, and stress. Laryngoscope 122, 196–203. doi: 10.1002/lary.22356, PMID: 21997855

[ref33] Palmer C. S. Niparko J. K. Wyatt J. R. Rothman M. de Lissovoy G. (1999). A prospective study of the cost-utility of the multichannel cochlear implant. Arch. Otolaryngol. Head Neck Surg. 125, 1221–1228. doi: 10.1001/archotol.125.11.1221, PMID: 10555693

[ref34] Pett M. A. (2015). Nonparametric Statistics for Health Care Research: Statistics for Small Samples and Unusual Distributions. United States: Sage Publications.

[ref35] Potts L. G. Litovsky R. Y. (2014). Transitioning From bimodal to bilateral Cochlear implant listening: speech recognition and localization in four individuals. Am. J. Audiol. 23, 79–92. doi: 10.1044/1059-0889(2013/11-0031), PMID: 24018578 PMC4195793

[ref36] Ramsden R. Greenham P. O'Driscoll M. Mawman D. Proops D. Craddock L.. (2005). Evaluation of bilaterally implanted adult subjects with the nucleus 24 Cochlear implant system. Otol. Neurotol. 26, 988–998. doi: 10.1097/01.mao.0000185075.58199.22, PMID: 16151348

[ref37] Robinson K. Gatehouse S. Browning G. G. (1996). Measuring patient benefit from otorhinolaryngological surgery and therapy. Ann. Otol. Rhinol. Laryngol. 105, 415–422. doi: 10.1177/000348949610500601, PMID: 8638891

[ref38] Sanchez-Cuadrado I. Lassaletta L. Perez-Mora R. Muñoz E. Gavilan J. (2015). Reliability and validity of the Spanish Glasgow benefit inventory after cochlear implant surgery in adults. Eur. Arch. Otorhinolaryngol. 272, 333–336. doi: 10.1007/s00405-013-2844-y, PMID: 24337876

[ref39] Schafer E. C. Amlani A. M. Paiva D. Nozari L. Verret S. (2011). A meta-analysis to compare speech recognition in noise with bilateral cochlear implants and bimodal stimulation. Int. J. Audiol. 50, 871–880. doi: 10.3109/14992027.2011.622300, PMID: 22103439

[ref40] Schafer E. C. Amlani A. M. Seibold A. Shattuck P. L. (2007). A Meta-analytic comparison of binaural benefits between bilateral cochlear implants and bimodal stimulation. J. Am. Acad. Audiol. 18, 760–776. doi: 10.3766/jaaa.18.9.5 18354885

[ref41] Services, U. D. O. H. A. H (2011). Foundation health measure report: health-related quality of life and well-being.

[ref42] Sladen D. P. Peterson A. Schmitt M. Olund A. Teece K. Dowling B.. (2017). Health-related quality of life outcomes following adult cochlear implantation: A prospective cohort study. Cochlear Implants Int. 18, 130–135. doi: 10.1080/14670100.2017.1293203, PMID: 28248612

[ref43] Sorrentino T. Donati G. Nassif N. Pasini S. Redaelli de Zinis L. O. (2020). Cognitive function and quality of life in older adult patients with cochlear implants. Int. J. Audiol. 59, 316–322. doi: 10.1080/14992027.2019.1696993, PMID: 31793801

[ref44] Spahr A. J. Dorman M. F. Litvak L. M. Van Wie S. Gifford R. H. Loizou P. C.. (2012). Development and validation of the AzBio sentence lists. Ear Hear. 33, 112–117. doi: 10.1097/AUD.0b013e31822c2549, PMID: 21829134 PMC4643855

[ref45] Straatman L. V. Huinck W. J. Langereis M. C. Snik A. F. M. Mulder J. J. (2014). Cochlear implantation in late-implanted Prelingually deafened adults: changes in quality of life. Otol. Neurotol. 35:253. doi: 10.1097/MAO.0b013e3182a4758e 24448285

[ref46] Vermeire K. Brokx J. P. Wuyts F. L. Cochet E. Hofkens A. Van de Heyning P. H. (2005). Quality-of-life benefit from cochlear implantation in the elderly. Otol. Neurotol. 26, 188–195. doi: 10.1097/00129492-200503000-00010 15793403

[ref47] Wackym P. A. Runge-Samuelson C. L. Firszt J. B. Alkaf F. M. Burg L. S. (2007). More challenging speech-perception tasks demonstrate binaural benefit in bilateral Cochlear implant users. Ear Hear. 28, 80S–85S. doi: 10.1097/AUD.0b013e3180315117, PMID: 17496654

[ref48] Wu Y.-H. Stangl E. Chipara O. Hasan S. S. Welhaven A. Oleson J. (2018). Characteristics of real-world signal to noise ratios and speech listening situations of older adults with mild to moderate hearing loss. Ear Hear. 39, 293–304. doi: 10.1097/AUD.0000000000000486, PMID: 29466265 PMC5824438

